# Thrombocytosis is a significant indictor of hypercoagulability, prognosis and recurrence in gastric cancer

**DOI:** 10.3892/etm.2014.1699

**Published:** 2014-04-29

**Authors:** CHANGYUAN HU, RENPIN CHEN, WENJING CHEN, WENYANG PANG, XIANGYANG XUE, GUANGBAO ZHU, XIAN SHEN

**Affiliations:** 1 Department of General Surgery, First Affiliated Hospital of Wenzhou Medical University, Wenzhou, Zhejiang 325035, P.R. China; 2 Department of Gastroenterology and Hepatology, First Affiliated Hospital of Wenzhou Medical University, Wenzhou, Zhejiang 325035, P.R. China; 3 Department of Microbiology and Immunology, Wenzhou Medical University, Wenzhou, Zhejiang 325035, P.R. China

**Keywords:** gastric cancer, thrombocytosis, recurrence, survival analysis

## Abstract

Although thrombocytosis has been reported in a variety of cancer types, the standard of thrombocytosis in gastric cancer (GC) and the association between thrombocytosis and the clinicopathological features of patients with GC remain unclear. In the present study, 1,763 GC patients were retrospectively filtered by preoperative thrombocytosis and compared with control group A (n=107) that had benign gastric lesions and control group B (n=100) that were GC patients with a normal platelet (PLT) count. Associations between clinical variables and preoperative PLT counts were assessed by univariate and multivariate analyses. Kaplan-Meier survival curves and Cox regression were used to evaluate the effect of thrombocytosis on prognosis. Sensitivities and specificities of the PLT counts in predicting recurrence were analyzed via area under the receiver operating characteristic curve (AUROC). The results indicated that the incidence of thrombocytosis in GC patients was higher than in benign gastric lesion patients, with 4.03% of GC patients having a PLT count >400×10^9^/l (P=0.014) and 12.08% had a PLT count >300×10^9^/l (P<0.001). For the patients with a PLT count >400×10^9^/l, the frequency of abnormal PLT counts in GC correlated with tumor size (P<0.001), tumor, node and metastasis (TNM) classification (P=0.002), invasive degree (P=0.003) and D-dimer (P=0.013) and fibrinogen concentrations (P=0.042). Tumor size (P=0.002), TNM classification (P<0.001) and depth of penetration (P=0.001) were independent factors for thrombocytosis. However, thrombocytosis functioned as an independent prognostic factor for GC patients with a PLT count >400×10^9^/l (relative risk, 1.538; 95% confidence interval, 1.041–2.271). In the majority of patients (17/24) with a high preoperative PLT count that decreased to a normal level following resection, PLT levels increased again at recurrence. Sensitivities and specificities of thrombocytosis for recurrence in those patients were 70.8 and 83.3%, respectively (AUROC, 0.847; P=0.01). Therefore, a PLT count of 400×10^9^/l is a suitable threshold for defining thrombocytosis in GC. Thrombocytosis was shown to affect the blood hypercoagulable state and also have a negative prognostic value for GC patients. PLT monitoring following surgery was useful to predict the recurrence for specific GC patients that suffered preoperative thrombocytosis but had restored PLT levels following resection.

## Introduction

Thrombocytosis can be induced by a number of primary and secondary factors. Primary thrombocytosis is a type of myeloproliferative disease with 50–70% of patients exhibiting mutations in the JAK2V617F gene and PLT levels >600×10^9^/l and even up to 1,000×10^9^/l (1). Secondary thrombocytosis is caused by drugs or other diseases, including inflammation, infection, rheumatism, anemia and malignancy, where patients exhibit moderately increased PLT levels that are between 400 and 800×10^9^/l (2–4). After excluding the diagnosis of essential thrombocythemia, preoperative cancer patients with a PLT count of >400×10^9^/l are considered to have secondary thrombocytosis (5–7). However, the threshold remains controversial.

Since the 1980s, the phenomenon that a comorbidity between blood hypercoaguable state and malignant solid tumors has caused widespread concerned and been increasingly studied. Although the specific mechanism is not completely understood, the association between thrombocythemia and the hypercoagulability state has been confirmed repeatedly (8). Prior to the evidence that malignant tumors lead to coagulation abnormality, Riess firstly proposed the symptoms of unexplained thrombocytosis in malignancies (9). Previously, thrombocytosis, as a paraneoplastic syndrome, has been successively identified in oral squamous cell, renal cell and hepatocellular carcinomas, as well as lung, esophageal, gastric (GC), colorectal, pancreatic and gynecological cancers (2,10–12).

Numerous studies agree that malignancies with unknown causes of thrombocytosis may be a valuable adjuvant parameter in predicting the prognosis of cancer (2,4,5,11). However, the complex effect of thrombocytosis in malignancy has been rarely reported. GC is the second most common malignancy in China, however, there are few specific early clinical manifestations and accurate predictors of prognosis. Therefore, GC patients with a high risk of recurrence may benefit from a reliable predicting indictor. In the present study, associations between thrombocytosis and clinicopathological features, survival time and tumor recurrence were systematically investigated in GC patients. The aim was to provide a new approach in the diagnosis and prognosis of GC.

## Methods and methods

### Definition of the blood test indicator

Primary thrombocytosis was eliminated using bone marrow cytomorphological examinations, and thrombocytosis was diagnosed as a PLT count >400×10^9^/l, which was in accordance with other studies (5,7,13). As the normal PLT count of patients at the First Affiliated Hospital of Wenzhou Medical College (Wenzhou, China) ranges between 100 and 300×10^9^/l, individuals with a PLT count >300×10^9^/l were also analyzed. Carcinoembryonic antigen (CEA), carbohydrate antigen 19-9 (CA19-9), D-dimer, fibrinogen, prothrombin time (PT) and activated partial thromboplastin time (APTT) standards were as follows: 0–5 μg/l; 0–37 U/ml; 0–0.5 mg/l; 2–4 g/l; 11–13 sec; and 30–45 sec, respectively. Sodium citrate anticoagulated blood samples were collected and analyzed with a COULTER Gen-S automatic blood analyzer (Beckman Coulter, Inc., Miami, FL, USA) and Stago STA-R Evolution automatic coagulometer (Diagnostica Stago, Inc., Beijing, China).

### Patients

A total of 1,763 patients who underwent surgical treatment for GC in the First Affiliated Hospital of Wenzhou Medical University between July 2005 and June 2008 were eligible for retrospective review in the study. None of the patients had received radiation therapy or chemotherapy prior to surgery, but had received strict chemical therapy following surgery, according to the National Comprehensive Cancer Network GC guidelines. Histopathological diagnosis of gastric adenocarcinoma was confirmed by the Pathology Department following surgery, according to the World Health Organization’s criteria. In total, 71 patients with GC and thrombocytosis were enrolled in study cohort A. These patients had a mean age of 63±9.15 years (range, 27–84 years). A total of 213 patients with a PLT count >300×10^9^/l were included in study cohort B and these individuals had a mean age of 63±10.02 years (range, 27–84 years). Control group A comprised 107 patients with benign gastric lesions that had a mean age of 41±6.33 years (range, 23–66 years). Control group B comprised 100 cases that had been randomly selected from 1,550 GC individuals with normal PLT counts. The mean age was 66±8.46 years (range, 34–88 years).

Blood samples were collected from the individuals within 1 week prior to surgery, ~4 weeks following surgery and during the follow-up period. B-mode ultrasound and physical examinations were performed postoperatively for patients with major complaints to assess the status of deep vein thrombosis (DVT). Imageological examinations, including computed tomography (CT) and enhanced CT scans, were performed to assess recurrence during the follow-up period. Informed written consent was provided by each patient and the study was approved by the Human Research Ethics Committee of the First Affiliated Hospital of Wenzhou Medical University.

### Statistical analysis

Associations between thrombocytosis and clinicopathological features were analyzed using the χ^2^ test, Fisher’s exact test, independent sample t-test, Pearson’s correlation test and logistic regression analysis. A Kaplan-Meier survival curve and Cox regression model were used to evaluate the clinical significance of thrombocytosis in GC. Area under the receiver operating characteristic curve (AUROC) and the Z-test were applied to analyze the sensitivities and specificities of PLTs in predicting recurrence. P<0.05 was considered to indicate a statistically significant difference. All analyses were performed using SPSS version 16.0 for Windows (SPSS, Inc., Chicago, IL, USA).

## Results

### Thrombocytosis in GC patients

As shown in Table I, when compared with control group A (benign gastric lesion, 0%, 0/107), the morbidity [4.03%, 71/1,763; 95% confidence interval (CI), 3.11–4.94] of GC patients with thrombocytosis (PLT >400×10^9^/l) increased significantly (P=0.014). This was also observed in patients with a PLT count >300×10^9^/l (12.08%, 213/1,763; 95% CI, 10.56–13.60; P<0.001).

### Correlation between thrombocytosis and clinicopathological features

As shown in Table II, the incidence of thrombocytosis, defined as a PLT count >400×10^9^/l, exhibited statistically significant differences when compared with the normal PLT cohort in tumor size (P<0.001), tumor, node and metastasis (TNM) classification (particularly for phase I, P=0.002) and depth of penetration (P=0.003). No statistically significant differences were identified in age, tumor location, type, degree of differentiation, vascular invasion, perineural invasion, lymphatic invasion, distant metastasis or tumor markers CEA/CA19-9 (P>0.05). The same result was verified in patients with a PLT count >300×10^9^/l with regard to tumor size (P=0.001), TNM classification (P<0.001) and penetration (P<0.001). Analysis of enumeration data revealed that no statistical significance existed between thrombocythemia and clinicopathological features in the cancer group (data not shown). In addition, to exclude the interaction between tumor size, type, differentiation, lymphatic invasion, penetration and TNM classification, risk assessments were used to measure the effect of these independent variables on thrombocytosis. Tumor size [P=0.002; odds ratio (OR), 2.179; 95% CI, 1.347–3.526], TNM classification (P<0.001; OR, 1.763; 95% CI, 1.317–2.360) and depth of penetration (P=0.001; OR, 1.643; 95% CI, 1.232–2.191) functioned as moderate positive factors for the occurrence of thrombocythemia.

**Figure 1:**
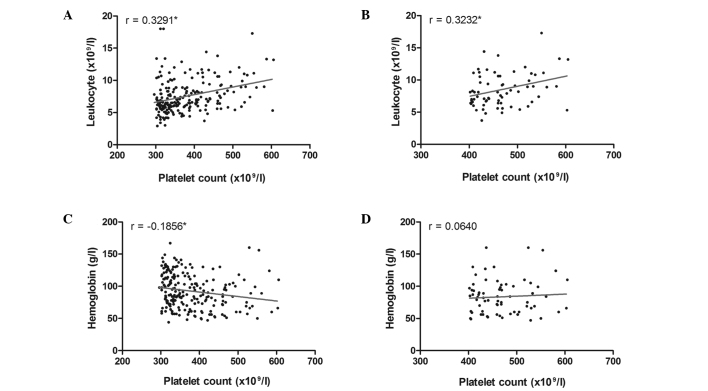
Linear correlation analysis. Correlations between leukocyte counts and (A) >300×10^9^/l (P<0.001) and (B) >400×10^9^/l (P=0.006) PLT counts. Correlation between hemoglobin concentration and (C) >300×10^9^/l (P=0.006) and (D) >400×10^9^/l (P=0.599) PLT counts. PLT, platelet.

The mean pretreatment PLT count for thrombocytosis patients was 469.23±53.99×10^9^/l (range, 402–605×10^9^/l), the leukocyte count was 8.54±2.61×10^9^/l (range, 3.7–17.3×10^9^/l) and the hemoglobin concentration was 83.51±26.68 g/l (range, 47–160 g/l). The mean PLT count for GC patients that had preoperative PLT levels >300×10^9^/l in study group B was 379.92±73.23×10^9^/l (range, 302–605×10^9^/l), while the leukocyte count was 7.81±3.95×10^9^/l (range, 2.9–20.6×10^9^/l) and the hemoglobin concentration was 92.61±27.14 g/l (range, 44–167 g/l). A significant positive linear correlation was identified between the PLT and leukocyte counts when the PLT count was >300 or 400×10^9^/l (r=0.3291; P<0.001; and r=0.3232; P=0.006, respectively; Fig. 1A and B). However, a correlation between the PLT count and hemoglobin concentration was only verified in patients with a PLT count >300×10^9^/l (r=−0.1856; P=0.006; Fig. 1C), since P=0.599 for patients with a PLT count >400×10^9^/l (Fig. 1D).

### Correlation between thrombocytosis and the blood hypercoagulable state

Although ultrasonic examinations did not reveal any statistically significant differences between the control group and study cohorts with PLT count >300×10^9^/l or 400×10^9^/l (P=0.444 and 0.083, respectively), DVT was more likely to affect tumor patients with thrombocytosis (7.04%, 5/71) than patients with a PLT count >300×10^9^/l (3.29%, 7/213). Abnormal D-dimer and fibrinogen concentrations occurred more frequently in patients with thrombocytosis (PLT>400×10^9^/l; P=0.004 and 0.013, respectively), but no statistically significant differences were observed when the threshold was defined as 300×10^9^/l. In addition, no correlation was observed between the occurrence of anomalous PLT counts and decreased PT/APTT (Table III). As aforementioned, the enumeration data of thrombocytosis demonstrated that no statistical significance was present between the cancer groups with regard to blood hypercoagulability (data not shown).

### Survival analysis in GC patients with thrombocytosis

**Figure 2:**
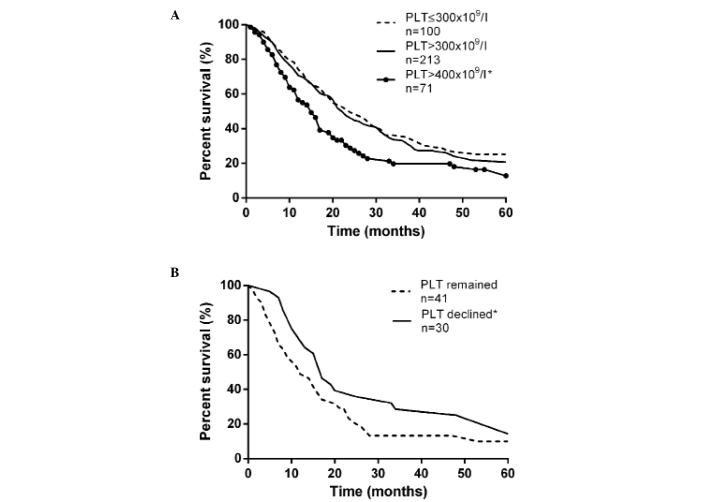
Survival analysis between GC patients with PLT counts that were (A) normal, >300×10^9^/l (P=0.227) and >400×10^9^/l (P=0.008) and (B) decreased to a normal level and remained >400×10^9^/l (P=0.417). PLT, platelet; GC, gastric cancer.

The overall 5-year survival rate of tumor patients with thrombocytosis (PLT >400×10^9^/l) was 16.90%, while the survival rate was 31.00% in individuals with a normal PLT count. Median survival times were 15 and 24 months, respectively (P=0.008, as determined by the logrank test; Fig. 2A). However, when compared with control group B, individuals with a PLT count >300×10^9^/l exhibited no significant difference in prognosis (P=0.227). The results demonstrated that ~42.25% (30/71) of patients did not have thrombocytosis following resection, however, the reason was unknown. Due to preoperative PLT counts being maintained at a high level in the most advanced-stage patients who were unable to undergo D2 dissection, the postoperative PLT count was also useful for predicting prognosis (P=0.046; Fig. 2B).

Prior to multivariate analysis, association analysis between thrombocytosis and clinicopathological features allowed the exclusion of age, location, type, vascular invasion and perineural invasion. Thus, PLT counts, TNM classification, depth of penetration, tumor size, degree of differentiation and lymphatic invasion were evaluated using the Cox proportional hazard model. Of the six factors, PLT count, TNM classification and lymphatic invasion were identified as independent prognostic indicators of survival (Table IV). Individuals with thrombocytosis had a relative risk (RR) for mortality of 1.538 (95% CI, 1.041–2.271; P=0.031), 1.994 for TNM classification (95% CI, 1.432–2.777; P<0.001) and 3.975 for lymphatic invasion (95%CI, 1.565–9.203; P=0.003).

### Thrombocytosis monitoring for the recurrence of GC

To evaluate the role of thrombocytosis (PLT >400×10^9^/l) in cancer recurrence, differences were compared between tumor patients with a normal PLT count and a group of individuals whose PLT count decreased from >400×10^9^/l to normal following surgery. Sensitivities for predicting the recurrence of malignancy in patients with normal and decreased PLT counts were 24.1 and 70.8%, respectively. In addition, the specificities of the two groups were 88.2 and 83.3%, respectively. Therefore, thrombocytosis in cancer patients with a decreased postoperative PLT count had a significant advantage for predicting tumor recurrence with AUROC, as compared with patients that had a normal PLT count prior to surgery (0.847 vs. 0.550; P=0.004; Table V).

## Discussion

As a multifunctional factor, an abnormal surplus of PLTs is associated with tumor size, TNM classification, invasive degree, prognosis and tumor recurrence in GC, as well as D-dimer and fibrinogen blood concentrations. The association between DVT and thrombocytosis remains to be clarified as ultrasonic testing was only performed on suspected patients that exhibited major complaints. In addition, the decrease in the number of PLTs temporarily following surgery may have been due to the surgical patients suffering blood loss during the procedure. Furthermore, clinical infusion during the fasting period contributed to hemodilution.

Malignant tumors with secondary thrombocythemia have been increasingly studied. Without a uniform standard, thrombocytosis has been defined as a PLT count >400×10^9^/l in the majority of studies (5–7). However, 220, 300, 350 and 500×10^9^/l have also been used as the threshold in previous studies (5,14–16). The confusion in the definition of thrombocytosis has caused deviation in experimental results and the reduction of lateral comparability between studies. As demonstrated in the present study, no statistically significant differences were identified between the two cancer groups with PLT counts of >300×10^9^/l and >400×10^9^/l with regard to the linear correlation between the PLT and leukocyte counts or the associated clinicopathological features. The difference in the linear correlation between hemoglobin concentrations and PLT levels was omitted due to the slight correlation. Survival analysis also illustrated the advantage of using PLT >400×10^9^/l as the standard for predicting prognosis. Clinically, there are a number of situations that can cause secondary thrombocytosis in patients with malignant solid tumors, including infection, anemia, inflammation and necrosis. Therefore, it is a reasonable hypothesis to define thrombocytosis in GC patients as those with a PLT count >400×10^9^/l. This excludes clinical confounding without causing a statistical deviation.

Although speculation remains, several hypotheses have been proposed with regard to the mechanisms by which thrombocytosis develops in malignancies. Released by the liver, kidney and skeletal muscle, thrombopoietin (TPO) specifically stimulates the proliferation and maturation of megakaryocytes, as well as the release of PLTs. In specific cases of tumor-associated thrombocytosis, the concentration of plasmatic TPO significantly increased due to complex pathophysiological factors (17). In addition, activation of the TPO receptor and the feedback regulation of TPO mRNA in the bone marrow participate in this phenomenon (18). However, TPO is not the only factor, as the body is able to produce a few PLTs following TPO gene knockout and the elimination of the TPO receptor (19). Interleukin-1 and -6 are bone marrow-stimulating cytokines that are associated with thrombocytosis and may potentially facilitate the production of PLTs (20,21). As previous studies have shown, the morbidity of thrombocytosis in various malignancies is inhomogeneous. It was found that ~42.5% of ovarian epithelial cancer cases presented with concurrent thrombocytosis, as well as 56.8% of renal cell carcinoma cases (2, 22). These results are much higher compared with other tumors, where the occurrence of thrombocytosis was 4.0% in GC patients and 4.5% in non-small-cell lung cancer patients (23). With regard to histoembryology classification, hematopoietic cells and the urogenital system originate from the same mesoderm, while the digestive and respiratory system originate from the same endoderm. Thus, we hypothesized that compared with the digestive system, the biological function of malignant tumors derived from the urogenital system may be more inclined to thrombocytosis.

Thrombocytosis may adversely affect survival in malignancies by promoting neoplasm invasion, adhesion and proliferation. Tumor cell-induced PLT aggregation may activate and aggregate PLTs to mediate adhesion to cancer cells via glycoproteins-Ib-IX, IIb/IIIa or adenosine diphosphate (24). PLTs efficiently shield and protect malignant cells from the host’s immune system and provide a useful medium for the adhesion of cancer cells to the vascular endothelium through forming tumor thrombi and adhesion molecules, including P-selectin and von Willebrand factor (25,26). Following adhesion, PLTs also play a role in tumor growth by secreting several tumor growth and angiogenic factors, including PLT-derived growth factor, arachidonic acid and vascular endothelial growth factor (VEGF). VEGF highly correlates with PLTs as an angiogenic factor and was shown to adversely affect survival in GC (27,28). Neovascularization is necessary for the development of neoplasm, which also explains the association between thrombocytosis and TNM classification, depth of penetration, tumor size and prognosis. However, predicting cancer recurrence with PLTs has been rarely reported. Although the mechanism is unclear, the clinical data indicate the significance of monitoring PLTs in a specific population; patients who suffered thrombocytosis preoperatively, but recovered following surgery.

In conclusion, a PLT count of 400×10^9^/l is an ideal threshold value for the definition of thrombocytosis. Although the incidence (4.0%) was lower than other types of cancer, thrombocytosis was shown to be associated with a number of clinicopathological features and function as an independent prognostic indicator and cancer recurrence monitor.

## Figures and Tables

**Table I:** Thrombocytosis in patients with gastric carcinomas or benign gastric lesions.

		PLT >300×10^9^/l		PLT >400×10^9^/l	
			
Group	PLT ≤300×10^9^/l, n	n	P-value	n	P-value
Gastric carcinoma	1,550	213	<0.001	71	0.014^a^
Benign gastric lesions	106	1		0	

a Calculated with Fisher’s exact test.

PLT, platelet. P-value compared the incidence of thrombocythemia with PLT count ≤300×10^9^/l in different groups.

**Table II:** Thrombocytosis and clinicopathological variables in GC.

		PLT >300×10^9^/l		PLT >400×10^9^/l	
			
Variable	PLT ≤300×10^9^/l, n	n	P-value	n	P-value
Age, years					
<60	39	75	0.516	24	0.488
≥60	61	138		47	
Location					
Upper third	20	31	0.082	10	0.339
Middle third	36	105		33	
Lower third	44	77		28	
Tumor size, cm					
<5	58	79	0.001	21	<0.001
≥5	42	134		50	
Type					
Adenocarcinoma	98	198	0.191^a^	67	0.263^a^
Squamous carcinoma	0	3		1	
Undifferentiated	2	12		3	
Degree of differentiation					
Well	15	19	0.242	10	0.886
Moderate	17	38		10	
Poor	66	156		48	
Vascular invasion					
Present	34	67	0.653	29	0.422
Absent	66	146		42	
Perineural invasion					
Present	37	80	0.924	26	0.959
Absent	63	133		45	
Lymphatic invasion					
Present	71	174	0.172	57	0.168
Absent	29	49		14	
TNM classification					
I	21	12	<0.001	1	0.002^a^
II	21	49		16	
III	44	105		39	
IV	14	47		15	
Depth of penetration					
T1	18	10	<0.001	1	0.003^a^
T2	4	15		5	
T3	53	97		37	
T4	25	91		28	
Distant metastasis					
Present	86	166	0.093	56	0.221
Absent	14	47		15	
CEA					
Abnormal	23	43	0.570	22	0.305
Normal	77	170		49	
CA19-9					
Abnormal	25	55	0.877	19	0.795
Normal	75	158		52	

a Calculated with Fisher’s exact test.

PLT, platelet; GC, gastric cancer; TNM, tumor, nodes, metastasis; CEA, carcinoembryonic antigen; CA19-9, carbohydrate antigen 19-9. P-value compared the incidence of thrombocythemia with PLT count ≤300×10^9^/l in different groups.

**Table III:** Thrombocytosis and coagulation markers in GC.

		PLT >300×10^9^/l		PLT >400×10^9^/l	
			
Variable	PLT ≤300×10^9^/l, n	n	P-value	n	P-value
PT					
Decreased	8	28	0.158	12	0.071
Normal	90	175		57	
APPT					
Decreased	11	30	0.465	13	0.204
Normal	83	172		56	
Fibrinogen					
Normal	83	158	0.061	51	0.042
Increased	15	52		20	
D-dimer					
Normal	87	172	0.172	51	0.013
Increased	13	41		20	
DVT					
Present	1	7	0.444^a^	5	0.083^a^
Absent	99	206		66	

a Calculated with Fisher’s exact test.

GC, gastric cancer; PLT, platelet; DVT, deep vein thrombosis; APTT, activated partial thromboplastin time; PT, prothrombin time. P-value compared the incidence of thrombocythemia with PLT count ≤300×10^9^/l in different groups.

**Table IV:** Multivariate analysis of the prognostic indicators.

Factors	RR	95% CI	P-value
PLT, ×10^9^/l			
≤300	1.000		
>400	1.538	1.041–2.271	0.031
TNM classification			
I	1.000		
II	1.692	0.544–5.267	0.364
III	3.339	0.941–11.852	0.062
IV	6.875	1.824–25.914	0.004
Depth of penetration			
T1	1.000		
T2	0.664	0.225–1.954	0.457
T3	0.856	0.447–1.641	0.640
T4	0.927	0.473–1.816	0.825
Lymphatic invasion			
Absent	1.000		
Present	3.795	1.565–9.203	0.003
Tumor size, cm			
<5	1.000		
≥5	0.826	0.555–1.228	0.344
Degree of differentiation			
Well	1.000		
Moderate	1.685	0.808–3.517	0.164
Poor	1.239	0.656–2.344	0.509

P-values were calculated using the Enter method. RR, relative risk; CI, confidence interval; TNM, tumor, nodes, metastasis; PLT, platelet.

**Table V:** Comparison of tumor recurrence among PLT levels.

		Sensitivities and specificities for recurrence					
		
Group	PLT, ×10^9^/l	Imaging	n	Sensitivity, %	Specificity, %	AUROC	P-value
Normal PLT (n=100)	>400	+	20				
		−	2				
	≤400	+	63			0.5500	
		−	15	24.1	88.2	95% CI, 408-0.691	0.521
Declined PLT (n=30)	>400	+	17				
		−	1				
	≤400	+	7			0.847^a^	
		−	5	70.8	83.3	95% CI, 0.707-0.988	0.010

a Declined PLT group had a significantly better AUROC value as compared with the normal PLT group (P=0.004).

AUROC, area under the receiver operating characteristic curve; PLT, platelet; CI, confidence interval. Imaging refers to computed tomography scans, +, recurrence is verified.
